# One year of SARS-CoV-2 and lung ultrasound: what has been learned and future perspectives

**DOI:** 10.1007/s40477-021-00575-x

**Published:** 2021-04-13

**Authors:** Andrea Boccatonda, Giulio Cocco, Eugenia Ianniello, Marco Montanari, Damiano D’Ardes, Claudio Borghi, Fabrizio Giostra, Roberto Copetti, Cosima Schiavone

**Affiliations:** 1 Division of Emergency Medicine, IRCCS Azienda Ospedaliero-Universitaria di Bologna, Bologna, Italy; 2 Internal Medicine, Department of Medicine and Science of Aging, ‘G. D’Annunzio’ University, Chieti, Italy; 3 Department of Medical and Surgical Sciences, University of Bologna, Bologna, Italy; 4 Emergency Department, Infermi Hospital, Rimini, Italy; 5 Emergency Department, Azienda Sanitaria Universitaria Friuli Centrale, Latisana General Hospital, Latisana, Italy; 6 Medicina d’Urgenza e Pronto Soccorso, Policlinico Sant’Orsola-Malpighi, Azienda Ospedaliero-Universitaria di Bologna, Via Pietro Albertoni, 15, 40138 Bologna, BO Italy

**Keywords:** Lung, COVID-19, SARS-CoV-2, Ultrasound, Imaging

## Abstract

A first screening by ultrasound can be relevant to set a specific diagnostic and therapeutic route for a patient with a COVID-19 infection. The finding of bilateral B-lines and white lung areas with patchy peripheral distribution and sparing areas is the most suggestive ultrasound picture of COVID-19 pneumonia. Failure to detect bilateral interstitial syndrome (A pattern) on ultrasound excludes COVID-19 pneumonia with good diagnostic accuracy, but does not exclude current infection. The use of shared semiotic and reporting schemes allows the comparison and monitoring of the COVID-19 pulmonary involvement over time. This review aims to summarise the main data on pulmonary ultrasound and COVID-19 to provide accurate and relevant information for clinical practice.

## Introduction

Since March 2020, the spread of SARS-CoV-2 infection has reached pandemic levels [1]. Notably, the infection progresses in an extremely heterogeneous way, passing from asymptomatic cases to patients requiring intensive care with severe respiratory failure [1, 2]. In the most serious cases, the infection leads to acute respiratory distress syndrome (ARDS)-like disease with diffuse alveolar consolidations (diffuse patchy-like lesions) [1, 3].

Several studies have underlined the importance of imaging in diagnosing COVID-19 [3, 4]. The typical computed tomography (CT) feature of a patient with acute COVID-19 infection is that of ground-glass opacities (GGO) or mixed GGO and consolidation and vascular enlargement; lesions are more likely to display a peripheral distribution and bilateral involvement and be lower-lung predominant [3, 4].

Given the need for the healthcare system to evaluate a huge number of patients with suspected COVID-19 infection, and to better manage resources and optimise therapy for patients in the right care setting, it is essential to employ a rapid execution diagnostic method that is both free of detrimental effects and contraindications and repeatable. Ultrasound meets all these requirements.

This review aims to summarise the main data on pulmonary ultrasound and COVID-19 to provide accurate and relevant information for clinical practice.

## Lung ultrasound execution scheme

Lung ultrasound is a method that is easy to execute and simple to learn [5]. Since it is a method based on the study of artifacts, any machinery can be sufficient, even without modern post-processing programs; moreover, its basic application does not require the use of color Doppler or contrast media [6–9]. Any probe can be employed, with the known limitations linked to the ultrasound physics characteristic of each probe. When referring to lung ultrasound, there is no unanimous consensus on the number of scans to be performed and in which lung fields [5]. In emergency setting, a rapid examination is preferred, characterised by a limited number of bilateral scans, generally 6 per hemithorax (2 anterior, 2 lateral and 2 posterior). In other settings, more scans can be performed to improve diagnostic accuracy, especially when searching for focal lung disease [5, 10, 11].

It is essential to consider the patient's position during the examination. In suspected cases of pneumonia, the posterolateral alveolar and/or pleural syndrome (PLAPS) point(s) is/are usually referred to as the elective area(s) in which to search [12–16]. The localisation of the specific infection undoubtedly depends on its pathogenetic mechanism (lobar bacterial pneumonia vs interstitial viral) [12–16]. In the specific case of SARS-CoV-2, the infection displays a typical non-homogeneous peripheral distribution [9, 17, 18].

**Fig. 1:**
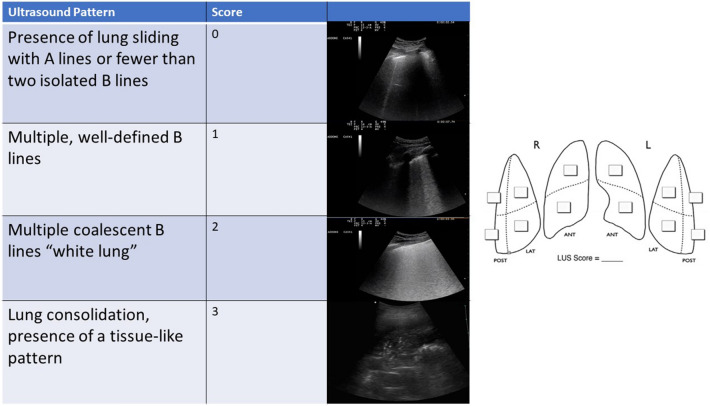
Lung ultrasound score execution scheme

Eventually, the use of a common scheme and a common ultrasound semeiotics allows for a serial follow-up over time, by evaluating changes in the sonographic features of lung fields examined [8, 9, 18, 19] (Fig. 1)

.

## Sonographic artifact evaluation

To understand the clinical significance of lung ultrasound artifacts, it is essential to know the pathophysiological basis of the disease. Recent data confirmed that the virus can induce cytopathic damage by binding to molecules such as ACE-2 and thus damaging pneumocytes [20, 21]. At the histological level, the infection first proceeds with an interstitial inflammatory infiltrate that ends up also progressively damaging the alveoli (alveolo-interstitial pneumonia). Furthermore, it produces evident endothelial damage and subsequent ‘capillaritis’ which is responsible for the generation of thrombosis and microembolism found in those patients [20, 21].

Therefore, changes of the subpleural interstitium are represented on ultrasound by the vertical artifacts arising from the pleural line, defined as B-lines [5]. Several suggestions and data in literature have described the peculiarities of the B lines in COVID-19 pneumonia. These often arise from an irregular pleural line, with minute subpleural consolidations, becoming wider while spreading in depth; moreover, there is often a lack of homogeneity between different lines, even in the same lung field [6, 7, 9, 18]. This is probably due to a relevant damage of the subpleural lung interstitium.

Therefore, COVID-19 pneumonia features on ultrasound have been described as ‘a storm of clusters of B-lines’ [7, 22].

An increasing number of B lines is related to a more relevant pathological change of the lung. When B lines completely occupy the lung field and become coalescent, it is called ‘white lung’; that feature has been often related to alveolar pulmonary edema in patients with heart failure [14, 18, 23]. In COVID-19 patients, preliminary data have shown that there is a correspondence between white lung on ultrasound and ground glass on HRTC [4, 6, 9].

The sonographic finding of ‘white lung’ is related to a serious histopathological change of the lung, with subsequent alveolar de-aeration. As the pathological process worsens with further air loss, lung parenchyma consolidates [4, 6, 9]. Therefore, subpleural consolidations with the presence of aerial bronchograms are found in the lung fields most severely damaged by the infectious process.

Almeida Monteiro et al. evaluated the agreement between sonographic image patterns and histological changes in ten fatal COVID-19 cases [10], and identified three distinct histological patterns: acute pulmonary injury, early fibroproliferative changes and a predominant pattern of fibroproliferation. Intriguingly, they demonstrated a full agreement between histological features and ultrasound images related to the ‘high-probability’ lung ultrasound pattern of COVID-19 [7–10].

In addition to the primary damage due to SARS-CoV-2 pneumonia, atelectasis areas, especially basal ones, due to poor ventilation stemming from muscle fatigue can also be highlighted in COVID-19 patients with severe respiratory failure [8, 9, 18]. The possibility of bacterial overinfection must also always be taken into consideration, as it can appear with an extended lung consolidation with dynamic air bronchogram [8, 9, 18].

Uncommon findings include anechoic pleural effusions, which were present in only 4.7% of patients in a work by Lomoro et al. [24] and in 10% of patients in another report [6].

Abolished pleural sliding with lung point can be detected in the case of pneumothorax (PNX) due to a very serious alteration of the lung parenchyma or mechanical ventilation barotrauma [9, 18, 25].

## Artifact distribution

**Fig. 2:**
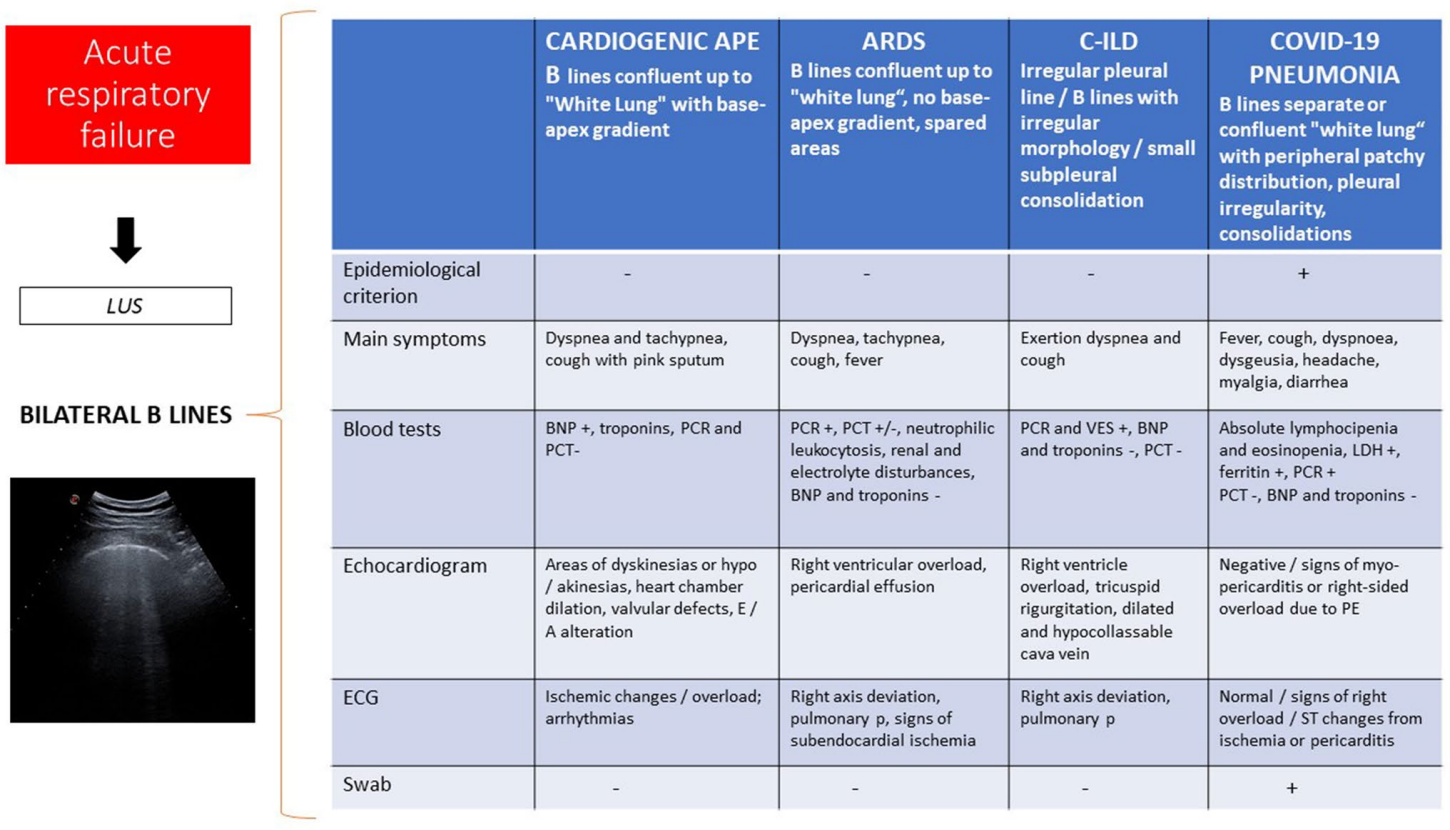
Differential diagnosis of interstitial syndrome based on epidemiological and clinical criteria

The study of artifacts’ locations in the lungs has always been a fundamental criterion for reaching a more precise etiological diagnosis. B lines, for example, are an expression of an interstitial syndrome, which in turn can be due to cardiovascular (cardiogenic edema), infectious-inflammatory (ARDS) or traumatic (contusion) diseases [10, 13, 14, 23, 26]. In the specific case of COVID-19 pneumonia, the changes are mainly peripheral, bilateral and not homogeneously distributed (patchy), with sparing areas [6–9, 18, 27]. In particular, severely altered lung areas (white lung) in mid-apical locations, but with basal sparing, can be high-suspicion findings for COVID-19 pneumonia [8]. Moreover, recent data reinforced the concept that SARS-CoV-19 pneumonia displays a bilateral patchy distribution of multiform clusters, alternating with ‘spared areas’, suggesting sonographic probability models for COVID-19 pneumonia based on ultrasound sign evaluation [7, 8, 18]. Those features allow the clinician to exclude a cardiogenic pulmonary edema with good accuracy. Furthermore, the ultrasound criterion integrated with the clinical and epidemiological ones enable a more specific diagnosis (Fig. 2).

## The role of pulmonary circulation

Thromboembolic disease is strongly associated to the pathophysiological process of COVID-19 pneumonia [18, 28, 29]. Some works reported a higher incidence of VTE in patients affected by SARS-CoV-2 pneumonia [18, 28, 29]. Small peripheral pulmonary embolisms are characterised on lung ultrasound by lesions with a clear morphology, with a pleural base, often with round or triangular morphology, in the context of few or absent B lines [23, 30]. It should be emphasised that pulmonary embolisms without peripheral venous thrombosis are often found in COVID-19 patients [30]. Indeed, endothelial/ microvascular damage of pulmonary capillaries seems to be the main trigger [20, 21, 31, 32]. The virus binds to pneumocytes via the ACE-2 receptor, thus leading to pneumocytic damage with activation of the inflammatory response and release of prothrombotic factors [20, 21, 31, 32]. Therefore, the finding of subpleural pulmonary consolidations with an ultrasound features compatible with that of an embolism must induce a strong clinical suspicion, especially in combination with a negative lower-limb CUS.

A recent work by Copetti et al. evaluated the sonographic features of pulmonary infarctions. Those are characterised by triangular hypoechoic consolidation with sharp margins, the absence of air bronchograms and a mostly central roundish hyperechoic area [33]. The authors hypothesised that air content within a pulmonary infarct represented the coexistence of aerated non-infarcted lung with the infarcted lung in the same lung area [33]. Therefore, they named that roundish hyperechoic area as the ‘survived lung’. This sonographic feature is related to the finding of bubbly consolidation on chest CT of patients with pulmonary infarction [33].

The reduction or absence of a vascular Doppler signal in peripheral or subpleural consolidation is often detected in COVID-19, and may represent peripheral segmental lung infarction due to microangiopathy, as evidenced by autopsy series [34].

A recent report by Tee et al. described CEUS employment to evaluate the physiopathology of subpleural ‘consolidations’. Indeed, avascular subpleural consolidation were most likely representing microinfarcts, whereas non-thrombotic consolidation displayed some enhancement [35].

## The contribution of the heart to the pathophysiology of COVID-19 infection

Cardiovascular changes play a relevant role in SARS-CoV-2 infection [36]. First, the development of pneumonia with alteration of the interstitium and pulmonary capillaries becomes a trigger of the aggravation and decompensation of pre-existing cardiac pathologies [18, 36]. Second, in some cases, the virus induces primary damage to the heart, with the development of myocarditis and pericarditis [18, 36]. Therefore, the overall evaluation of a COVID-19 patient, especially one with severe respiratory failure, cannot disregard a cardiac ultrasound evaluation with the aim of evaluating the kinetics of the cardiac chambers in search of segmental and global alterations, the evaluation of the right heart and of the inferior vena cava with estimation of the pressures, and the search for anechoic liquid at the pericardial level [18, 36–38]. It is relevant to evaluate cardiac right chamber pressures (tricuspid regurgitation) and inferior cava vein collapsibility index, thus calculating pulmonary arterial pressure (PAPs), as they are influenced by the primary damage of the virus (capillaritis), by possible pulmonary embolisms and by the application of external pressures in mechanical ventilation [18, 32, 36–38].

## Lung ultrasound on invasive mechanical ventilation patients

Lung ultrasound can guide pulmonary recruitment and pronation maneuvers in patients undergoing invasive ventilation; indeed, ultrasound can identify atelectatic lung areas (parenchymal consolidations without aerial artifacts), which demonstrate an alveolar re-expansion following the setting of high PEEP values, as underlined by the reappearance of a subpleural aerial interface [9, 18]. Furthermore, the evaluation of diaphragmatic motility can be an index of lung compliance [9, 18]. Previous works have already shown how diaphragm ultrasound can be predictive of a successful extubating attempt through the calculation of derived parameters [39–43].

## Evidenced-based data

In 2009, Testa et al. evaluated the accuracy of lung ultrasound during H1N1 pandemic influenza A [44]. They performed lung ultrasound screening in an emergency department looking for the presence of interstitial syndrome, alveolar consolidation, pleural line abnormalities and pleural effusion; a sensitivity of 94.1%, a specificity of 84.8%, a positive predictive value of 86.5% and a negative predictive value of 93.3% were detected for the diagnosis of H1N1 pneumonia [44].

In 2020, Poggiali et al. reported their experience in the role of lung ultrasound by evaluating 12 COVID-19 patients in an emergency department setting [17]. In all the patients, authors found diffuse B pattern with spared areas, while posterior subpleural consolidations were detected in only three patients [17]. Ground-glass opacity on chest CT scan showed a strong correlation with a bilateral B pattern on ultrasound [17].

Another study from two medical centers in China compared LUS and chest CT performed on COVID-19 patients [27]. Results confirmed that COVID-19 lesions are more often detected on posterior lung fields with bilateral distribution [27]. By comparing the detection of B lines, consolidations and PE on a total of 540 lung regions, authors argued that LUS is more sensitive than chest CT in the diagnosis of regional alveolar-interstitial pattern (60% vs. 38.5%), alveolar-interstitial syndrome (93.3% vs. 68.9%), consolidation (38.9% vs. 3%) and PE (74.4% vs. 15.6%) [27].

Lu and colleagues performed a retrospective evaluation of 30 patients with SARS-CoV-2 infection [45]. Results showed that interstitial pulmonary edema (90.0%) and pulmonary consolidations (20.0%) are main features in COVID-19 patients, and that lung lesions are mainly present in the subpleural and peripheral pulmonary fields, in particular on the lower lobe and in the dorsal region [45]. There was moderate agreement (*κ* = 0.529) between lung ultrasound and chest CT [45]. The sonographic scores to evaluate mild, moderate and severe lung lesions showed sensitivity levels of 68.8%, 77.8% and 100.0%; specificity levels of 85.7%, 76.2% and 92.9%; and diagnostic accuracy levels of 76.7%, 76.7% and 93.3%, respectively [45]. Therefore, authors concluded that the diagnostic efficacy of lung ultrasound is relatively low for mild to moderate patients, but high for severe ones [45].

## Need for a diagnostic algorithm and future directions

The SARS-CoV-2 pandemic has changed and is changing the medical care system [1, 2, 37]. The development of diagnostic tests and therapeutic strategies is constantly evolving. In the first phase of the pandemic, attention was focused on the diagnosis of COVID-19 pneumonia in a context of high pre-test probability following contact with infected subjects and in any case in a context of widespread of the virus. Therefore, the ultrasound finding of bilateral interstitial lung disease in patients reporting contact with a COVID-19 + subject and suggestive symptoms were sufficient to make a diagnosis and to prescribe therapy. Few works focused on the follow-up of the infected patient or on ultrasound findings as a healing criterion. A diagnostic gold standard is currently lacking, and the diagnosis can only be confirmed with good certainty if the swab is positive. However, the nasopharyngeal swab is characterised by non-optimal sensitivity, but absolute specificity [47]. As mentioned above, the infection progresses clinically in an extremely heterogeneous way; therefore, not all infected patients develop pneumopathy. A negative ultrasound exam can only rule out COVID-19 pneumopathy, but it does not exclude infection or the contagiousness of a patient [18, 47]. That aspect represents a main concern regarding the hospitalisation criteria, especially in terms of choosing the right care setting for patients (COVID-19 free, suspicious or COVID-19 wards).

Generally, in the case of a patient with suspected infection, if the swab and lung ultrasound are not suggestive, the patient can be admitted to a non-COVID-19 ward with good safety [18].

**Fig. 3:**
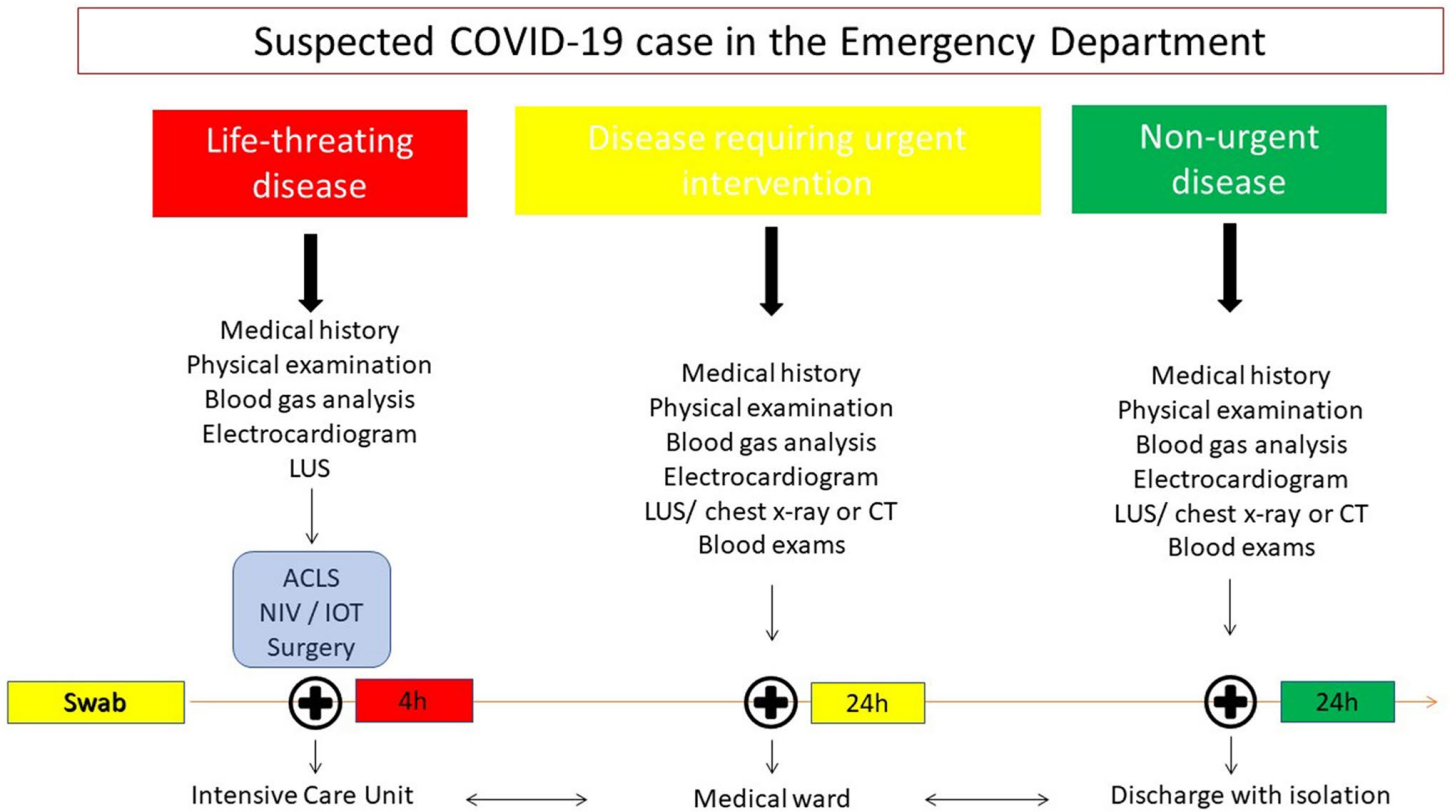
Suggested algorithm to manage COVID-19 patients in the emergency department

In the emergency department, where the patient is evaluated for the first time by medical staff, ultrasound seems to find its main role (Fig. 3). The physician who evaluates a patient in emergency setting, thus having to make life-saving decisions and manoeuvres in a few hours or minutes, will not be able to immediately know the swab result, and in some cases, the critical condition of the patient does not allow the physician to perform chest CT. Therefore, ultrasound becomes the main imaging criterion to ascertain any COVID-19 interstitial pneumonia to integrate with the medical history, physical examination, electrocardiogram and blood gas analysis. At the same time or after stabilising the patient, a nasopharyngeal swab with high priority (e.g., 4 h) will be performed, and the result will allow the patient to be directed to a COVID-19 ward or elsewhere.

In other situations where life-saving interventions are not required, it is possible to wait for the result of the swab; in those cases, ultrasound becomes the first-level imaging method, to be integrated eventually with other diagnostic/instrumental assessments. The ultrasound finding of bilateral A pattern can reasonably rule out COVID-19 interstitial pneumonia, while a positive finding can lead to a request for a CT scan if necessary.

Therefore, the medium-to-low-intensity suspected COVID-19 patient may be admitted and managed in an intermediate ward (COVID-19 suspected) to perform a second swab at 24–48 h or a BAL, or non-COVID ward, depending on the epidemiological, clinical and imaging (ultrasound ± chest CT) findings. Finally, the COVID-19 patient can be discharged form hospital if clinical conditions are stable, with recommendations of home isolation and activating the public health service for monitoring. However, the detection of sonographic signs compatible with COVID-19 pneumonia is linked to a greater clinical impairment of the patient, and, therefore, to a higher probability of hospitalisation.

The data currently present in the literature, especially those found in the few trials or prospective works performed, have pointed out the diagnostic accuracy of thoracic ultrasound.

Indeed, the specificity of the ultrasound signs correlated with SARS-CoV-2 infection is often not sufficient to confirm the diagnosis alone, thus leading to misdiagnosis [46]. Moreover, it needs to be highlighted that lung ultrasound cannot detect lesions deep within the lung parenchyma [47].

**Fig. 4:**
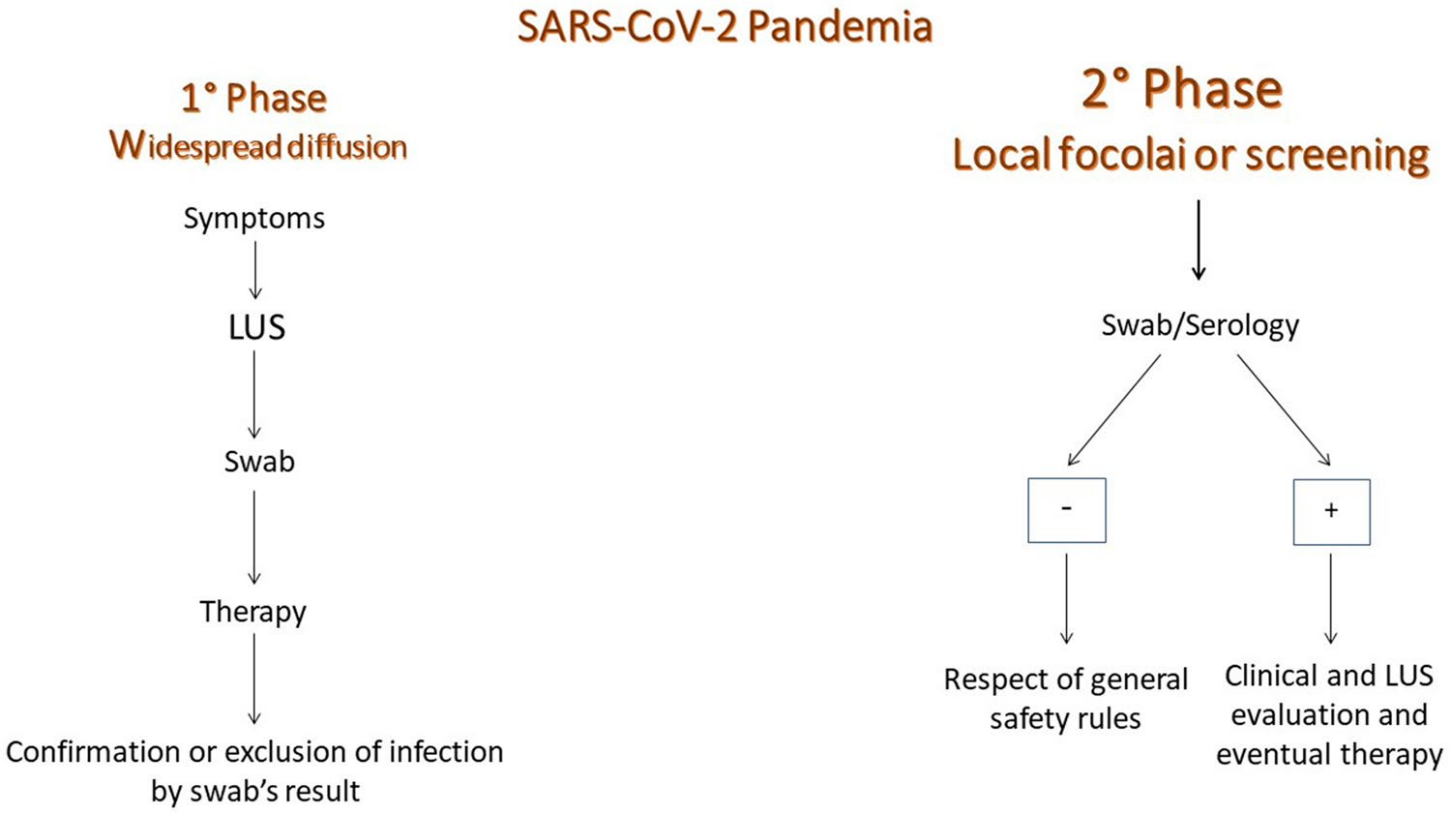
Role of lung ultrasound in different phases of the COVID-19 pandemic

**Fig. 5:**
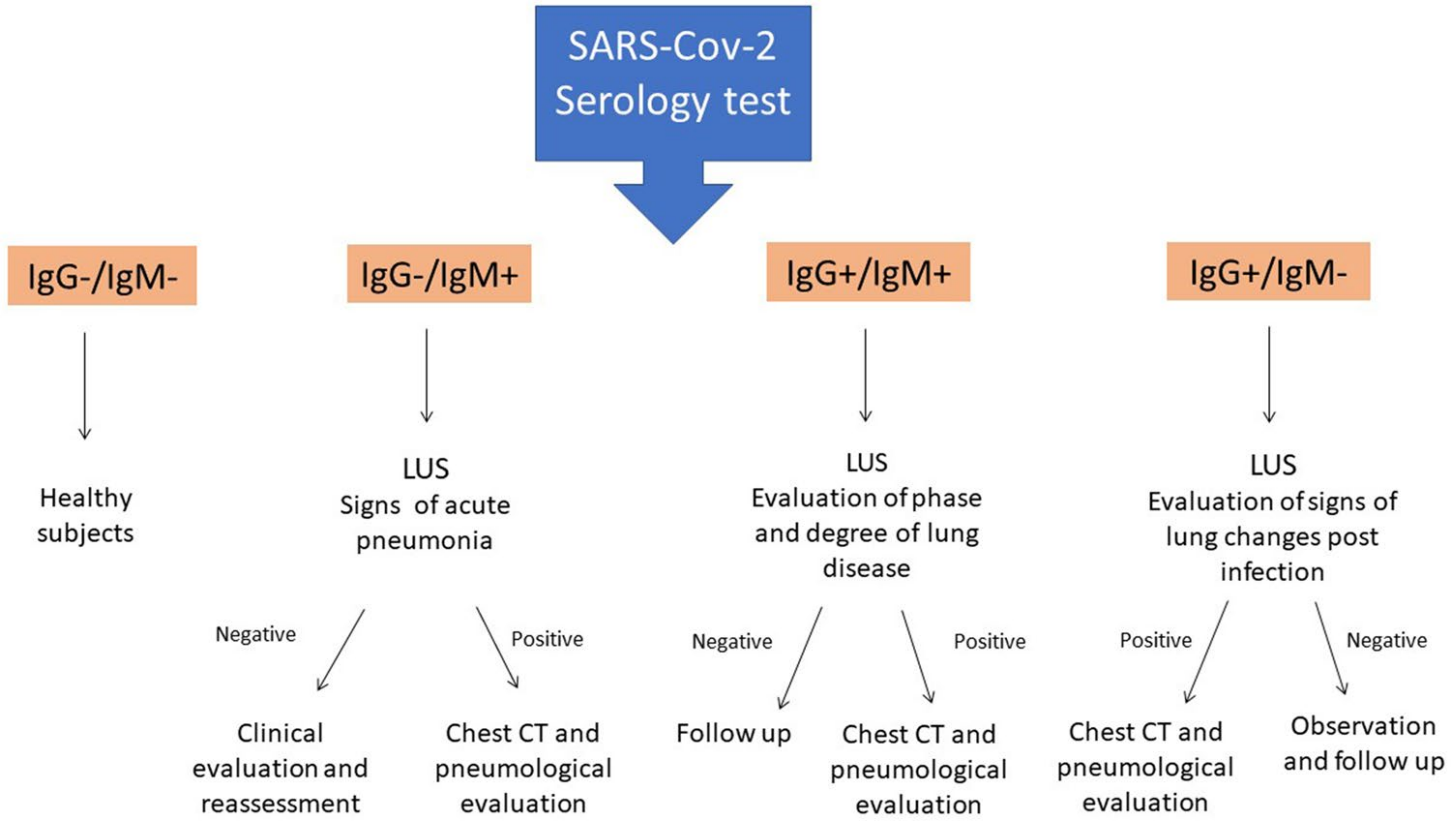
Role of lung ultrasound and COVID-19 serology

Currently, new scenarios and management concerns are coming to light. How to evaluate a patient with new onset symptoms in which the outcome of the swab is not immediately available? How to evaluate disease progression in affected patients? Can ultrasound play a role in defining the healing of a COVID-19 sufferer? Lung ultrasound should probably be used in this new phase as an immediate screening tool, which can lead to a faster diagnosis and to early activation of public health surveillance to limit the spread of the infection (Fig. 4). Furthermore, given the introduction of larger-scale screening strategies (swab and serological tests), the physician performing lung ultrasound will be responsible for evaluating possible lung involvement in patients in different clinical stages of the disease. In those cases, lung ultrasound can evaluate signs of acute or subacute lung involvement or lung pathological changes after clinical recovery (Fig. 5).

## Conclusions

- Given the extreme heterogeneity of the clinical manifestation, and considering that many of the symptoms are common to other pathologies, a first screening with a portable/pocket-sized ultrasound by the family doctor or emergency physician can be relevant to set a specific diagnostic and therapeutic route for the patient, even in an outpatient setting, thus preventing overcrowding in emergency rooms and clinics.
- Lung ultrasound is the first imaging method in emergency setting to be integrated with clinical data, ECG and blood gas analysis.
- The finding of bilateral B-lines and white lung areas with patchy peripheral distribution and sparing area, is the most suggestive ultrasound picture of COVID-19 pneumonia.
- Failure to detect bilateral interstitial syndrome (A pattern) on ultrasound excludes COVID-19 pneumonia with good diagnostic accuracy, but does not exclude current infection.
- Lung ultrasound is characterised by excellent sensitivity, especially in the emergency department setting, but poor specificity towards the diagnosis of interstitial pneumonia from COVID-19 [18].
- The use of shared semiotic and reporting schemes allows the comparison and monitoring of the COVID-19 pulmonary manifestation over time.
- Although the data in the literature show that COVID-19 pneumonia has a prevalent peripheral involvement, it should not be forgotten that lung lesions that do not reach the pleural surface cannot be visualised in ultrasound due to ultrasound physics.
- In some cases, SARS-CoV-2 infection induces relevant cardiovascular alterations; therefore, it is suitable to complete a point-of-care evaluation at the cardiac level, looking for anomalies of the kinetics or pericardial effusions.
- COVID-19 is linked to a high incidence of pulmonary embolism and deep vein thrombosis; therefore, the clinician must always maintain a high suspicion towards this complication by looking for pulmonary ultrasound signs (triangular subpleural consolidations—bubble sign), vascular (CUS) and cardiac (increase in right ventricle and non-collapsible inferior cava vein).
- The epidemiological change of the infection and the introduction of large-scale screening methods may enhance the role of lung ultrasound as a complementary tool in the assessment of lung damage.
